# Notes and Comments

**Published:** 1899-04

**Authors:** 


### Notes and Comments.[1]

Journalistic Changes.—In the March number of the *Dental Brief,* Dr. T. B. Welch announces his relinquishment of its editorial management. After many years of faithful and conscientious work Dr. Welch lays down the pen that has proved so helpful to his many readers, both morally and professionally.

With the April number of the same magazine, Professor Wilbur F. Litch assumes editorial control. The new incumbent is so well known in the dental world, both as a teacher and writer, that he will receive a most hearty welcome from every quarter. It is with pleasure that we welcome him to the ranks, and feel assured that we shall all receive much stimulus from his able pen. Our best wishes go to the new editor of the *Dental Brief,* and we congratulate the publisher upon securing the services of so eminent a man.

---

We regret the loss of two of our contemporaries, the *American Dental Weekly* and the *Dental Practitioner and Advertiser.* We were always glad of the weekly visits of the former, and looked forward with interest to the time for the arrival of Dr. Barrett's quarterly. It is hoped some avenue may be opened that we may continue to hear from both these energetic editors.

---

"Information."—This neat, home dental magazine, conducted by Dr. L. P. Bethel, has just made its fifth visit. It is breezy and filled with good and interesting material. We bespeak for it a successful and most valuable career. If this magazine could be placed in the schools and public libraries, it would no doubt do much towards solving the difficult problem of educating the public on dental matters.

---

[1] The assistant editor solicits contributions for this department,—new methods, new remedies and formulas, or any short practical note which may prove of value to the practitioner or student. Address 1338 Walnut Street, Philadelphia.

Professional Recruits.—What Dr. Taft writes we always read with interest. His experience and standing in the profession entitle his opinions to much consideration. But we cannot but be a little amused at his schemes for providing homes for the sixteen hundred annual recruits to dental ranks, with an increasing ratio. It will be seen that the doctor is laying hold of the recently acquired territory for homes for some of them, and he goes to foreign fields with others, while he decimates the veterans at the rate of four hundred and eighty per annum.

There is no doubting the fact that the multiplying of colleges is rapidly multiplying the ranks of the profession. With the good doctor's figures, we will have to acquire more territory before many years to find homes for our recruits.—Editorial in *Western Dental Journal.*

---

The Egotist.—I suspect a dentist of being shallow that knows it all, that replies to every suggestion, "That's nothing new; I know a better way than that," and is always ready to show you what he can do. He may be smart, but he is certainly egotistic, and he is probably so self-opinionated that he deprives himself of much he could learn by being more teachable. The wisest of us can be taught something; the most skilful can get new points, and none of us know it all.

At our conventions we can generally discern the most learned and skilful by their teachableness, simplicity of manners, and the modesty of their statements.—Dr. T. B. Welch.

